# Supplementary figures and images for: In Silico and In Vitro Analysis of MAP3773c Protein from Mycobacterium avium subsp. Paratuberculosis
Source: Biology (Basel). 2022 Aug 6;11(8):1183. doi: 10.3390/biology11081183 (PMC9405291; doi:10.3390/biology11081183)

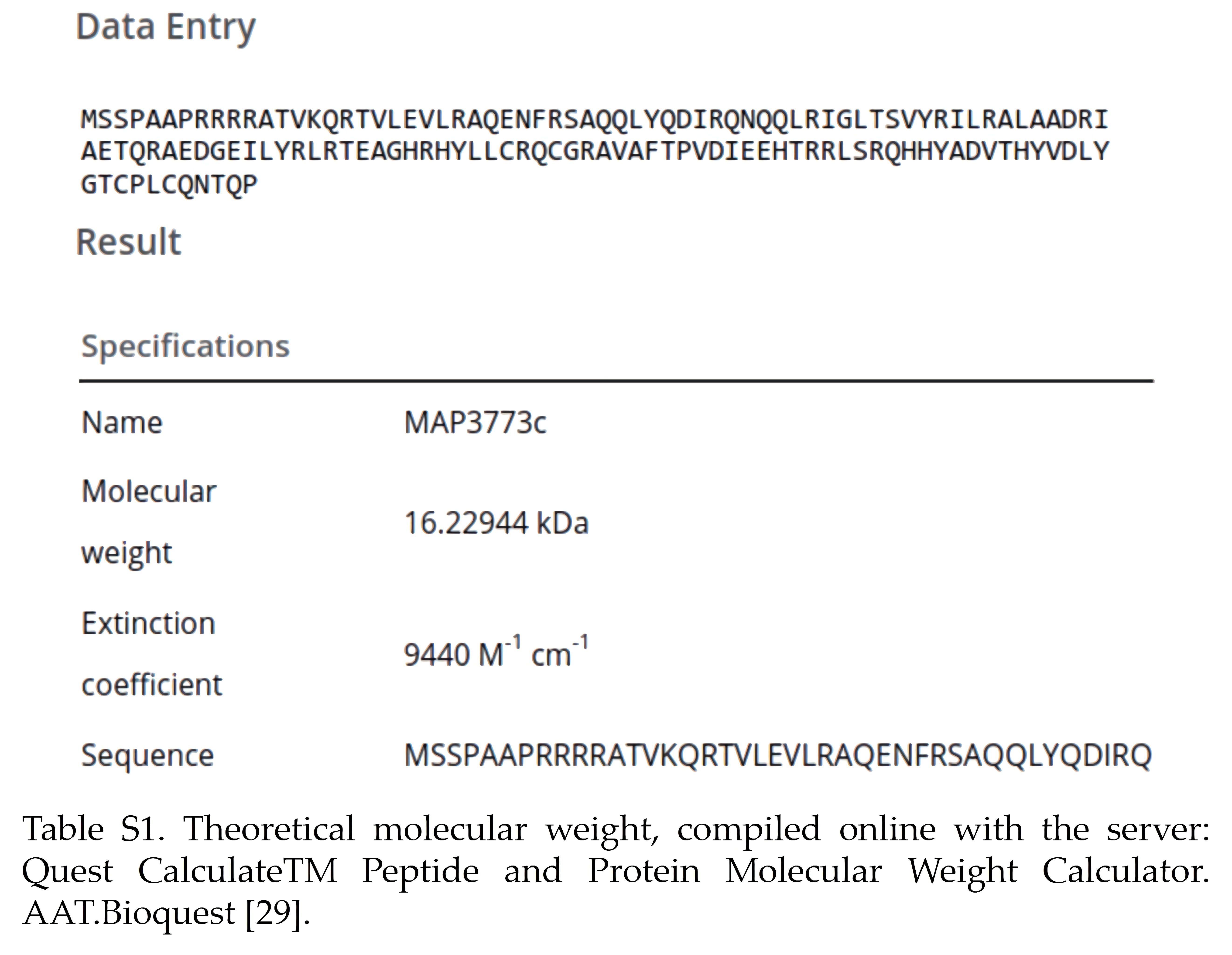

Supplement: Supplementary file 1 [file biology-11-01183-s001.zip › biology-1778505-supplementary.jpg]
